# Prevalence and Associated Factors of Coexistence of Malnutrition and Sarcopenia in Geriatric Rehabilitation

**DOI:** 10.3390/nu13113745

**Published:** 2021-10-23

**Authors:** Shinta Nishioka, Tatsuya Matsushita, Anna Yamanouchi, Yuka Okazaki, Kana Oishi, Emi Nishioka, Natsumi Mori, Yoshiharu Tokunaga, Shinya Onizuka

**Affiliations:** Nagasaki Rehabilitation Hospital, 4-11, Ginya-machi, Nagasaki 8500854, Japan; wkymf348@yahoo.co.jp (T.M.); s.y.k.o@outlook.jp (A.Y.); okazaki@zeshinkai.or.jp (Y.O.); dh-reha@zeshinkai.or.jp (K.O.); nrh.dietitian@zeshinkai.or.jp (E.N.); nutrition@zeshinkai.or.jp (N.M.); tokunaga@zeshinkai.or.jp (Y.T.); onizuka@zeshinkai.or.jp (S.O.)

**Keywords:** older adults, malnutrition, sarcopenia, rehabilitation

## Abstract

Malnutrition and sarcopenia often coexist in rehabilitation patients, although they are often overlooked and undertreated in clinical practice. This cross-sectional study aimed to clarify the prevalence of the coexistence of malnutrition and sarcopenia (Co-MS) and its associated factors in convalescent rehabilitation wards in Japan. Consecutive patients aged ≥ 65 years in convalescent rehabilitation wards between November 2018 and October 2020 were included. Malnutrition and sarcopenia were determined by the Global Leadership Initiative on Malnutrition (GLIM) criteria and the Asian Working Group for Sarcopenia (AWGS 2019) criteria, respectively. Patients who presented both with malnutrition and sarcopenia were classified as Co-MS. Potentially associated factors included age, sex, days from onset to admission of rehabilitation wards, reason for admission, pre-morbid functional dependency, comorbidity, activities of daily living, swallowing ability, and oral function and hygiene. The prevalence of malnutrition, sarcopenia, and Co-MS was calculated. Binary logistic regression analyses were performed to compute odds ratios (ORs) and the 95% confidence interval (CI) of possible associated factors for each condition. Overall, 601 patients were eligible for the analysis (median 80 years old, 355 female patients, 70% cerebrovascular disease). Co-MS, malnutrition, and sarcopenia were found in 23.5%, 29.0%, and 62.4% of the enrolled patients, respectively. After adjustment, onset–admission interval (OR = 1.04; 95% CI = 1.02 to 1.06), hospital-associated deconditioning (OR = 4.62; 95% CI = 1.13 to 18.8), and swallowing ability (Food Intake LEVEL Scale) (OR = 0.83; 95% CI = 0.73 to 0.93) were identified as independent explanatory factors of Co-MS. In conclusion, Co-MS was prevalent in geriatric rehabilitation patients; thus, healthcare professionals should be aware of the associated factors to detect the geriatric rehabilitation patients who are at risk of both malnutrition and sarcopenia, and to provide appropriate treatments.

## 1. Introduction

Malnutrition and sarcopenia are conceptually different conditions, but often overlap in geriatric rehabilitation patients [1,2]. Malnutrition is defined as “a lack of intake or uptake of nutrition that leads to altered body composition and body cell mass resulting in diminished function and impaired outcome” by the European Society for Clinical Nutrition and Metabolism [1]. Indeed, decreased nutritional status is possibly associated with poor functionality or functional recovery in patients undergoing rehabilitation [3,4,5,6]. A meta-analysis showed that 13% (95% confidence interval [CI]: 5–20) and 47% (95% CI: 40–54) of older rehabilitation patients were malnourished and at risk of malnutrition, respectively [7]. On the other hand, sarcopenia is defined as a progressive and generalised skeletal muscle disorder associated with an increased risk of adverse outcomes [2]. It is more common than malnutrition, with a prevalence of 40–76% according to the definition by European or Asian working groups [8,9,10]. In addition, sarcopenia is associated with worse recovery of activities of daily living (ADLs) and swallowing function in geriatric rehabilitation patients [8,9,10]. Thus, early detection and treatment for both conditions are urgent issues to maximise functional capacity and quality of life of geriatric patients.

The aetiology of sarcopenia is associated with disease burden, inappropriate nutritional intake, and inactivity in addition to ageing, and it shares the same feature as malnutrition, which is a loss of muscle mass [2]. Consequently, the coexistence of malnutrition and sarcopenia (Co-MS) is prevalent and is receiving growing interest [11,12,13,14]. In an acute care setting, 4.9% of the patients had Co-MS, and it was associated with higher mortality in hospitalised older patients than malnutrition or sarcopenia alone [12]. Additionally, only two studies investigated the prevalence of Co-MS in geriatric rehabilitation patients [13,14]. A Spanish study conducted in a post-acute care unit showed that 14.8% of older patients had Co-MS [13]. Another study that examined Australian geriatric rehabilitation inpatients indicated that 23%, 1.3% and 13% of the malnourished patients had probable sarcopenia, non-severe sarcopenia, and severe sarcopenia, respectively [14]. Increasing awareness of Co-MS should be prioritised, because these two conditions are often overlooked and undertreated in clinical practice and have aforementioned adverse effects on mortality [12,15,16].

However, no study investigating the prevalence of Co-MS has been conducted in a rehabilitation setting for an Asian population. The Global Leadership Initiative on Malnutrition (GLIM) proposed lower cut-off values of body mass index (BMI) for Asian patients to diagnose malnutrition than those for non-Asian patients [17]. Similarly, in 2019, the Asian Working Group on Sarcopenia (AWGS) established the definitions for sarcopenia specific for the Asian population (AWGS 2019 criteria) [18]. The AWGS 2019 criteria diagnose sarcopenia when the patients have low muscle mass (e.g., skeletal muscle mass index of <7.0 kg/m^2^ for men and <5.7 kg/m^2^ for women based on the bioimpedance analysis) and low handgrip strength (<28 kg for men and <18 kg for women) or poor physical performance (e.g., 6 m walk test < 1.0 m/sec) [18]. On the other hand, the criteria proposed by the European Working Group for Sarcopenia in Older People (EWGSOP2 criteria) diagnose sarcopenia when older adults exhibit low handgrip strength (<27 kg for men and <16 kg for women) and low muscle mass (appendicular skeletal muscle mass index < 7.0 kg/m^2^ for men and <6.0 kg/m^2^ for women) [2]. Thus, the prevalence of Co-MS in Asian geriatric rehabilitation patients may be different from other ethnicities. Clarifying the prevalence of the overlap between both conditions in the Asian population would be helpful to increase awareness of Co-MS in healthcare professionals.

Additionally, the factors related to Co-MS have not been reported. Previous studies have shown that age, sex, reason for admission, days from disease onset to hospital admission to rehabilitation, activities of daily living (ADL), premorbid ADL, and swallowing ability were associated with malnutrition [19,20]. Additionally, age, comorbidity, ADL, and oral factors were associated with sarcopenia [9,21]. Although all of the above factors are also expected to be associated with Co-MS, there is no information for these factors to date. Exploring the factors related to Co-MS will be useful to detect the geriatric patients at risk of malnutrition and sarcopenia. 

Therefore, this study aimed to describe the prevalence of Co-MS using the consensus-based criteria and to examine its possible associated factors.

## 2. Materials and Methods

### 2.1. Study Design and Participants

This study comprised a cross-sectional analysis using the dataset of the retrospective cohort study conducted in three convalescent rehabilitation wards in a hospital in Nagasaki, Japan (the original cohort study that aims to examine the effects of Co-MS on the functional outcomes is now under submission). The convalescent rehabilitation ward is a unique system that provides an inpatient rehabilitation service for up to a maximum 3 h per day and up to 180 days consecutively by a multidisciplinary team involving medical doctors, nurses, physical therapists, occupational therapists, speech-language-hearing therapists, registered dietitians, care workers, social workers, dental hygienists, and pharmacists [22]. Consecutive patients aged 65 years or older between November 2018 and October 2020 were included. All patients were transferred from acute care hospitals. Patients who were unable to undergo muscle mass measurement, or were not assessed for it within 7 days from admission, who had a pacemaker implanted, or whose hospital fees were not covered by public healthcare insurance (≥60 days elapsed from disease onset to admission or stayed ≥ 180 days in the convalescent rehabilitation wards) were excluded. The reasons for admission were classified into cerebrovascular disease (e.g., stroke, traumatic brain injury), orthopaedic disease (e.g., hip fracture, vertebral compression fracture), and hospital-associated deconditioning. All data, including demographic characteristics, were collected from medical records or the database containing the specific data (e.g., nutritional status) directory recorded by healthcare professionals (e.g., registered dietitian) within the hospital.

This study was conducted in accordance with the principles of the Declaration of Helsinki and received approval from the local ethics committee (approval number: R2–16). Informed consent was waived as this study only relied on the anonymised data from daily clinical practice. We provided an opt-out option to allow patients to withdraw their participation from this study. 

### 2.2. Assessment of Malnutrition

Malnutrition was defined using the GLIM criteria [17], which comprise two steps: screening risk of malnutrition using a validated tool and fulfilling both phenotypic and aetiologic criteria. The risk of malnutrition was screened using the Malnutrition Universal Screening Tool (MUST) [23]. MUST comprises a weight loss score, BMI score, and acute illness score: the total scores range from 0 to 6 [23]. A score of 0, 1 and ≥2 indicates no, moderate, and high risk of malnutrition, respectively. Thus, a MUST score of ≥1 was regarded as having a risk of malnutrition in this study. The MUST has been validated for Japanese patients with pulmonary tuberculosis [24]. Therefore, although MUST has not been validated for Asian patients in a rehabilitation setting, it could be applied to the population in the current study. Trained registered dietitians performed all the GLIM procedures including the MUST. Patients with MUST scores of ≥1 were subjected to phenotypic and etiologic criteria assessment.

Phenotypic malnutrition criteria include low body mass index (BMI), unintentional loss of body weight, and low skeletal muscle mass index (SMI) (Table S1). Low BMI and low SMI were defined using Asia-specific cut-off values: low BMI was defined as <18.5 (aged < 70 years) or <20 kg/m^2^ patients (aged ≥ 70 years); low SMI was defined as <7.0 (males) or <5.7 kg/m^2^ (females) (Table S1) [17,18]. Height was determined using a stadiometer or a measuring tape (if the patients could not stand alone), while body weight was measured using calibrated scales which allowed measuring while on a wheelchair. Both measurements were performed by nursing staff on the day of admission. The registered dietitians determined the degree and duration of weight loss as part of a comprehensive nutritional assessment. Skeletal muscle mass was determined by means of bioimpedance analysis (BIA) using Inbody s10 instrumentation (Inbody Japan, Inc., Tokyo, Japan). After an interval of one hour or more following a meal, patients took at least five minutes of rest. The patients were then asked to lie in a supine position before BIA measurement was performed by trained physical therapists.

Aetiologic criteria for malnutrition were divided into “decreased food intake/assimilation” and “disease burden/inflammation” (Table S1). The severity of malnutrition was confirmed using these ethnicity-specific cut-off values: low BMI for mild/moderate malnutrition and severe malnutrition were defined as 17.0–18.5 kg/m^2^ (patients aged < 70 years) or 17.8–20 kg/m^2^ (patients aged ≥ 70 years), and <17.0 kg/m^2^ (patients aged < 70 years) or <17.8 kg/m^2^ (patients aged ≥ 70 years), respectively; low SMI for mild/moderate malnutrition and severe malnutrition were 5.2–7 kg/m^2^ (males) or 4.8–5.7 kg/m^2^ (females), and <5.2 kg/m^2^ (males) or <4.8 kg/m^2^ (females), respectively (Table S1) [17,25,26]. Since no specific cut-offs for severe loss of muscle mass existed, severely decreased skeletal muscle mass was defined as four standard deviations below the mean of healthy adolescents, based on previous studies in Japanese [26,27]. 

Aetiologic criteria for malnutrition were assessed by means of an interview with the patients or their representative, information from the acute care hospital regarding nutritional intake, primary disease (i.e., reason for admission) and comorbidities, laboratory testing for inflammatory response (e.g., C-reactive protein), and/or diagnosis by the medical doctor. Because the GLIM criteria did not establish any criteria for “disease burden/inflammation”, our study did not set specific criteria and cut-off values for inflammatory markers. The registered dietitians who assessed the GLIM criteria were trained in diagnosing malnutrition by a senior dietitian at least 6 months prior to the study. This training included enhancing their clinical judgement in the presence of a disease burden/inflammation. Severe traumatic brain injury, multiple traumas, and any other disease/injuries which require intensive care are examples of acute illness with inflammation we inferred. On the other hand, cancer, chronic obstructive pulmonary disease, and chronic heart failure with elevated inflammatory status (e.g., elevated C-reactive protein) are examples of chronic disease with inflammation.

### 2.3. Assessment of Sarcopenia

One researcher (T.M.) diagnosed sarcopenia using low SMI and low handgrip strength based on the AWGS 2019 criteria [18,28]. Another author subsequently double-checked the diagnosis of sarcopenia by confirming the raw data on skeletal muscle mass and handgrip strength (S.N.). Low skeletal muscle mass was identified when SMI < 7.0 kg/m^2^ for males and <5.7 kg/m^2^ for females [18]. Physical therapists measured handgrip strength using a Smedley-type hand dynamometer grip D (Takei Scientific Instruments, Niigata, Japan). Patients were directed to sit with one arm extended vertically. The cut-off values used for low handgrip strength were <28 kg and <18 kg for males and females, respectively [18]. To maximise the generalisability to the population of interest, we included the patients for whom handgrip strength could not be measured due to cognitive impairment or hemiparesis. Patients for whom we were unable to measure handgrip strength were deemed as having a handgrip strength of “0 kg”; thus, they were automatically classified as having low handgrip strength. Furthermore, we did not perform a physical performance test because many patients could not be assessed due to physical disabilities.

### 2.4. Coexistence of Malnutrition and Sarcopenia

The patients who fulfilled the criteria for malnutrition (regardless of the severity) as well as for sarcopenia were classified as having Co-MS. 

### 2.5. Patient Characteristics and Factors Potentially Associated with Co-MS

Patient characteristics, including age, sex, onset–admission interval, and type of nutrition care (i.e., oral intake, enteral nutrition, or combination of both), were collected from the medical records.

The comprehensive rehabilitation team assessed the ADLs using the Functional Independence Measure (FIM) [29]. The FIM includes thirteen motor domains (i.e., eating, grooming, bathing, dressing upper body, dressing lower body, toileting, sphincter control, bladder management, bowel management, transfer to bed/chair/wheelchair, transfer to toilet, transfer to tub/shower, mobility with walk/wheelchair, stair-climbing) and five cognitive domains (i.e., comprehension, expression, social cognition, social interaction, problem solving, memory), which evaluates the ADLs performed in daily life. Each domain was scored from 1 (totally de-pendent) to 7 (independent). Subsequently, the overall score total from 18 to 126 (motor FIM: 13 to 91, cognitive FIM: 5 to 35) was also computed. A higher FIM score indicates a greater ability to perform ADLs.

The Food Intake LEVEL Scale (FILS) is a 10-grade scale to assess any swallowing dysfunction [30], and was evaluated by speech-language-hearing therapists (Table S2). The FILS scores are based on the swallowing condition and can be scored subjectively ranging from level 1 (not performing swallowing training due to severe dysphagia or unconsciousness) to level 10 (no problem with eating). Levels 1 to 3, 4 to 6, and 7 to 10 indicate “no oral intake” (i.e., the patients do not take any food due to severe dysphagia or unconsciousness), “oral intake and alternative nutrition” (i.e., the patient intake food orally in addition to enteral or parenteral nutrition) and “oral intake alone”, respectively [30]. It has been validated with the Functional Oral Intake Scale and showed significant correlation (r = 0.96–0.99) [30].

The Revised Oral Assessment Guide (ROAG) was used by trained dental hygienists to evaluate oral hygiene and oral functions [31]. Components of ROAG include voice, swallowing, lips, teeth/dentures, mucosa, gingiva, tongue, and saliva. Each item was scored from 1 (good) to 3 (severe disability), while the total score ranged from 8 to 24. Total scores of 8, 9 to 12 and 13 to 24 mean “normal oral status”, “mild to moderate oral problems” and “severe oral problems”, respectively [31]. The details of the ROAG are described in Table S3.

We evaluated functional dependency before the admission to acute care hospital (“pre-morbid functional dependency”) using certification of long-term care insurance [32]. Patients who were certificated as “long-term care” (level 1 to 5, based on the degree of dependency) were identified as having pre-morbid functional dependency. Care levels are defined by the Japanese public long-term healthcare insurance policy: care level 1 status is requiring assistance for some aspect of daily activities (e.g., housekeeping) for around 25–32 min/day, whereas care level 5 status is needing assistance for almost all activities (e.g., eating, toileting) for 110 min/day or over. This status can be regarded as a surrogate marker for functional dependency before admission to an acute care hospital, and have been used in previous studies [3,10,19,20]. 

The effect of comorbidities was evaluated using the updated version of Charlson comorbidity index (CCI) collected from electrical medical charts recorded by medical doctors [33]. Updated CCI is scored if the patients have the following comorbidities other than the diseases that were reason for admission: congestive heart failure (2 points); dementia (2 points); chronic pulmonary disease (1 point); rheumatologic disease (1 point); mild liver disease (2 points); diabetes with chronic complications (1 point); hemiplegia or paraplegia (2 points); renal disease (1 point); any malignancy, including leukaemia and lymphoma (2 points); moderate or severe liver disease (4 points); metastatic solid tumour (6 points); and AIDS/HIV (4 points). The total score ranges between 0 and 24 [33]. 

### 2.6. Statistical Analyses

Numerical variables with normal distribution were expressed using the mean (SD), whereas those with skewed distribution were presented using the median (interquartile range). Normality was confirmed using histograms. For categorical variables, numbers (percentage) are shown. Crude odds ratios (ORs) of each associated factor for malnutrition, sarcopenia, Co-MS were separately calculated. Additionally, adjusted ORs of all potentially associated factors were computed using binary logistic regression analysis. We selected the following potentially associated factors using the available evidence and physiological plausibility: age, sex, onset–admission interval (i.e., days between the onset of acute illness/injuries and admission into a convalescent rehabilitation ward), pre-morbid functional dependency, reason for admission (cerebrovascular disease, orthopaedic disease, and hospital-associated deconditioning), FIM (both motor and cognitive scores), FILS, ROAG, and CCI [4,9,10,19,20,21]. All factors were entered into binary logistic regression models for each outcome using the data on all patients, regardless of availability of handgrip strength measurement. Numerical variables were entered as continuous variables into regression models. For the reason for admission, cerebrovascular disease was used as a reference. To perform sensitivity analysis for the association between the potentially associated factors and Co-MS, we also implemented binary logistic regression analysis for the patients who were able to measure handgrip strength. Patient data with missing values were excluded from the analyses that used those missing values, but were included in the analyses using variables that were not missing. For example, the patients with missing values of the FIM were excluded from the comparison of FIM between the groups, but were included in the analyses of other variables (e.g., age, sex). Statistical significance was established at ≤5%.

## 3. Results

**Figure 1:**
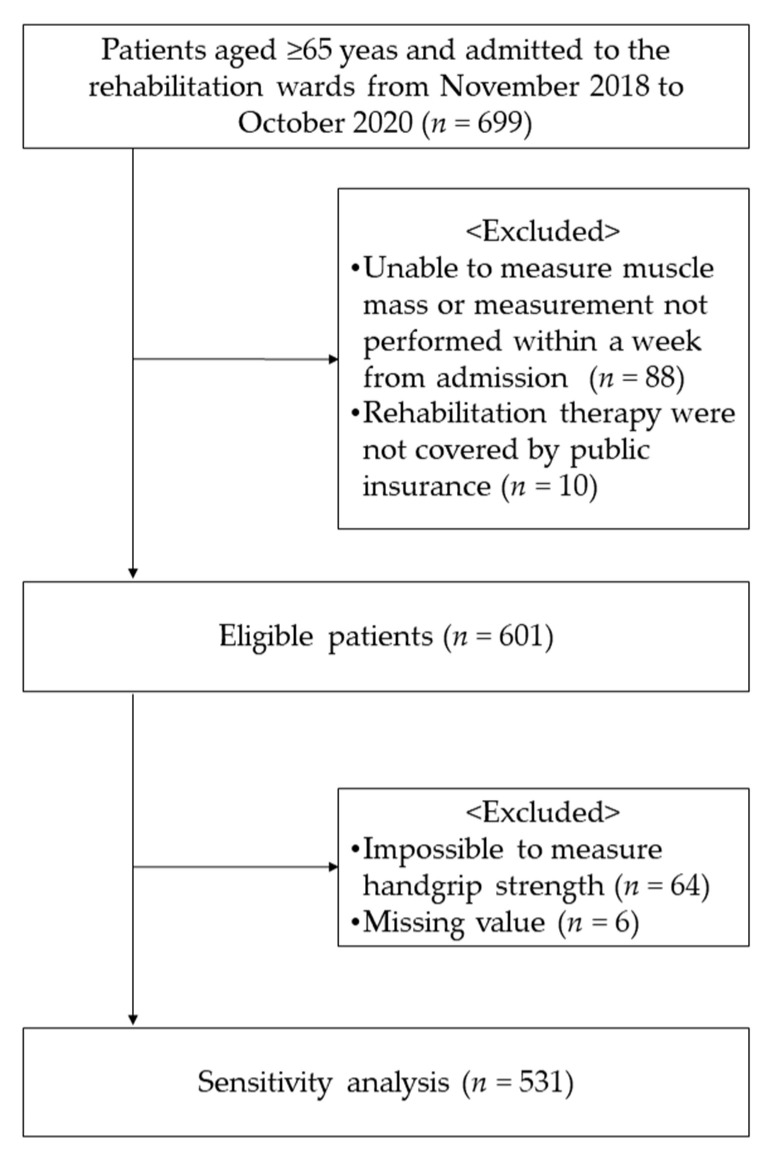
Flowchart of selection for study participants.

A total of 699 patients aged 65 years or older were identified during the study period. Among these, 88 patients who were unavailable to undergo a valid muscle mass measurement within seven days from admission and 10 patients who were not covered by healthcare insurance were excluded. Finally, 601 patients (median 80 years old, 246 males [40.9%] and 355 females [59.1%]) were eligible for this study (Figure 1). Of these, 13.6% (82/601) of the patients were aged < 70 years. 

Table 1 shows the demographic and clinical characteristics of the study participants. Among the patients, approximately 70% of the patients were admitted due to cerebrovascular disease, followed by orthopaedic disease (27.3%) and hospital-associated deconditioning (2.0%). Functional dependency before the onset of primary disease or injury (i.e., reason for admission) was found in nearly a quarter of the patients. Median FIM score (indicating around four points for each lower-order item) suggested that at least half of the patients need assistance in performing ADL (e.g., food intake, walking and transferring to bed). Around half of the patients had one or more comorbidities (CCI score of ≥1). Additionally, almost all patients (97%) had oral health problem based on the ROAG score. Most patients could take food orally. However, a few patients (7.8%) received total enteral nutrition.

**Figure 2:**
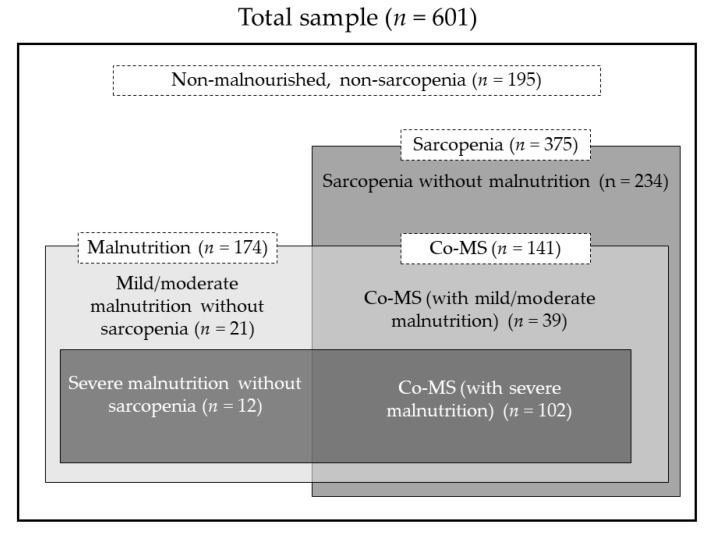
Overlapping of malnutrition and sarcopenia. Co-MS: Coexistence of Malnutrition and Sarcopenia.

Table 2 and Table S4 show the prevalence of malnutrition, sarcopenia, and Co-MS in the study participants. Overall, Co-MS was found in 23.5% (141/601) of the patients (25.6% of male patients and 22.0% of female patients). The prevalence of malnutrition and sarcopenia was 29.0% (174/601) and 62.4% (375/601), respectively. The SMI and handgrip strengths in males were significantly higher than those in females (mean SMI for males and females; 6.4 kg/m^2^ vs. 4.9 kg/m^2^, *p* < 0.001: median handgrip strength for males and females; 24.6 kg vs. 13.7 kg, *p* < 0.001). Regarding the phenotypic criteria of malnutrition, low SMI was most likely found in the study samples (39.3%). Among the patients with MUST scores of ≥1, approximately 90% had low SMI (236/261). Twenty-eight percent of the patients showed decreased food intake or assimilation, whereas 5.8% indicated inflammation as an aetiology of malnutrition. As shown in Figure 2, 81.0% of the patients with malnutrition were concurrently defined as Co-MS (141/174). Of these, 72.3% (102/141) had severe malnutrition. On the other hand, the patients with sarcopenia were less likely to have Co-MS (37.6%, 141/375). 

In multiple logistic regression analysis, six patients were excluded due to missing values for pre-morbid functional dependency; thus, 595 patients were included (Table 3). After adjustment, longer onset–admission interval, hospital-associated deconditioning, and lower FILS scores were independent explanatory factors for Co-MS. Older age and lower FIM-motor domain score also showed a tendency for an association with increased risk of Co-MS, but this was not significant. Explanatory factors for malnutrition (longer onset–admission interval and lower FILS score) were different from those for sarcopenia (older age, lower FIM motor and cognitive scores).

Table S5 indicates crude ORs for malnutrition, sarcopenia, and Co-MS. Among the potentially associated factors, age, onset–admission interval, hospital-associated deconditioning, pre-morbid functional dependency, CCI, and ROAG scores were associated with higher odds for Co-MS at a 5% level of significance. On the contrary, FIM (both motor and cognitive domains) and FILS were inversely associated with the odds for Co-MS.

For sensitivity analysis, 64 patients for whom handgrip strength could not be measured and 6 patients with missing values for pre-morbid functional dependency were excluded: thus, 531 patients were included for the analysis (Table 4). Binary logistic regression analysis showed that longer onset–admission interval (OR = 1.04, 95% CI = 1.02 to 1.06) and lower FILS (OR = 0.80, 95% CI = 0.69, 0.92) were independently associated with Co-MS (R^2^ = 0.19). On the other hand, unlike the overall case analysis, hospital-associated deconditioning was not associated with Co-MS. Factors related to malnutrition and sarcopenia were the same as the overall case analysis, except for FIM-motor and FIM-cognitive for sarcopenia. 

## 4. Discussion

The results of our cross-sectional analysis reveal that malnutrition and sarcopenia coexisted in 23.5% of the geriatric rehabilitation patients when using consensus-based criteria. Moreover, potentially associated factors for the coexistence of malnutrition and sarcopenia were a longer onset to admission interval, hospital-associated deconditioning, and poor swallowing function as indicated by FILS.

### 4.1. Prevalence of the Coexistence of Malnutrition and Sarcopenia 

The prevalence of the Co-MS in our study was higher than that in a Spanish study conducted on a post-acute geriatric rehabilitation unit with a smaller sample size and used the EWGSOP criteria for sarcopenia and ESPEN diagnosis criteria for malnutrition (*n* = 88, 14.8%) [13]. However, the prevalence in our study was nearly identical to an Australian study which investigated the geriatric rehabilitation of inpatients (*n* = 506, 23%), and employed EWGSOP2 criteria and GLIM criteria for sarcopenia and malnutrition, respectively [14]. Since the EWGSOP2 criteria uses the same criteria (low SMI and low handgrip strength) as the AWGS 2019 criteria, the results of the Australian study can be comparable to ours. To the best of our knowledge, this is the first study to clarify the prevalence of Co-MS in Asian rehabilitation patients. In addition, our results suggest that Co-MS is prevalent regardless of race.

The proportion of Co-MS in geriatric rehabilitation patients was slightly higher compared with that of patients in acute care hospitals (4.6–12%) [12,34,35]. Higher prevalence suggests that geriatric rehabilitation patients have a greater tendency to be exposed to potential causes of both malnutrition and sarcopenia such as progressive inflammation, decreased food intake, and inactivity. However, as the definition of sarcopenia and malnutrition varied across the studies in acute care hospitals [12,34,35], further studies employing the up-to-date criteria for both malnutrition and sarcopenia are needed.

As the Asian population has a lower BMI than a non-Asian population, the contemporary definition of malnutrition involved ethnicity-specific cut-off values for BMI [2,7]. The definitions of sarcopenia are also proposed by the European and Asian working groups separately [2,18]. Although our study did not examine the appropriateness of the race-specific cut-off values for BMI or SMI, race-specific criteria might be helpful to compare the prevalence of malnutrition, sarcopenia and Co-MS. 

### 4.2. Potentially Associated Factors of the Co-MS

In this study, a longer period until admission to rehabilitation wards was associated with Co-MS. One possible explanation for this is that a prolonged stay in an acute care hospital could be associated with the onset of malnutrition. Patients with more severe disease or those with multiple complications tend have the length of their hospital stay extended. Acute and severe inflammatory response may promote muscle catabolism and increase metabolic demands that would be compensated by gluconeogenesis from muscle tissues, leading to nutritional deterioration [36]. Contrarily, the onset–admission interval was not associated with sarcopenia in this study. The reason for the inconsistence between malnutrition and sarcopenia may be explained by the variety of rehabilitation provision in acute care hospitals. A previous study showed that 10 days of bed rest resulted in 3.2% muscle mass loss and the loss of 15.6% of the isokinetic muscle strength [37]. Since some patients with a longer stay in an acute care hospital received rehabilitation to regain muscle function, a longer hospital stay did not necessarily mean longer periods of disuse. Some proposed approaches to mitigate muscle mass loss in an acute care setting include physical rehabilitation, neuromuscular electrical stimulation, or early mobilisation strategy [38]. However, as previously reported, it is also possible that the patients who had malnutrition and/or sarcopenia before the disease onset tend to have a longer length of stay [39,40]. Thus, whether Co-MS in rehabilitation patients can be prevented by specific approach in acute care setting remains to be studied.

Another associated factor for Co-MS was poor swallowing function. It is clinically plausible that because patients with dysphagia often present with decreased food intake, they result in having malnourishment more often compared to non-dysphagic patients [41]. Contrarily, our results do not show an association between swallowing ability and sarcopenia. These can be explained by the fact that the majority of our patients (70.7%) were diagnosed with cerebrovascular disease. Recent studies suggested that there is a causal relationship between sarcopenia and dysphagia, which is known as sarcopenic dysphagia [42]. However, patients with diseases that basically cause dysphagia (e.g., stroke) were excluded from the diagnosis of sarcopenic dysphagia [42]. Contrarily, muscle atrophy due to hemiparesis may play a potential role in causing sarcopenia in stroke patients [43]. Thus, sarcopenia can be developed in cerebrovascular patients regardless of swallowing ability. As previous studies showed a relationship between decreased muscle mass, the onset of dysphagia and poor recovery of swallowing function [44,45], the association between dysphagia and sarcopenia would be strengthened in patients without cerebrovascular disease.

Our sensitivity analysis showed almost identical results from the overall case analysis, and thus the results are robust. The only exception was the association of hospital-associated deconditioning. The reason why the deconditioning did not show a significant association with Co-MS can be attributed to a substantially small number of deconditioning patients in the sensitivity analysis (*n* = 10), supported by wide confidence interval (OR = 2.98, 95% CI = 0.65 to 13.6). Previous studies showed that the patients with hospital-associated deconditioning had a high prevalence of malnutrition and sarcopenia: >90% of the deconditioning patients had malnutrition and sarcopenia after pneumonia [9]; 88% of the deconditioning patients in an acute care hospital showed malnourishment [46]. Therefore, the association of hospital-associated deconditioning for Co-MS would be stronger if the number of patients increased. An alternative explanation for the inconsistency is that some hospital-associated deconditioning patients had missing values for hand-grip strength and the rest of the patients had a relatively stronger grip strength. However, all of the deconditioning patients included in the sensitivity analysis (*n* = 10) had low hand-grip strength; therefore, this hypothesis is unlikely.

All numerical variables (e.g., FIM, FILS, ROAG, CCI) were entered as continuous ones into multiple logistic regression analysis. This may be the reason why the ORs for these variables were relatively small (around 1). If the categorical variables (e.g., presence or absence of dysphagia) were employed for the regression analyses, the ORs would be greater than the obtained values. Indeed, the crude OR of sarcopenia for dysphagia (both dichotomised variables) was reported as being 6.17 [47]. Additionally, occlusal support (an indicator of oral function) showed 4.00 of OR for nutritional improvement [48].

Our findings may be generalised for Asian geriatric rehabilitation patients. Several studies conducted in Asian societies reported factors associated with malnutrition and sarcopenia that are similar to the results of our study. A systematic review, which included a study conducted in rehabilitation facilities in Singapore, showed that dysphagia was associated with malnutrition [41]. Additionally, onset–admission interval can be associated with a risk of weight loss in patients in convalescent rehabilitation wards [49]. Regarding sarcopenia, older age and limited mobility were independently associated with decreased muscle mass in hospitalised older adults in Japan [50].

### 4.3. Limitation

Our study has several limitations. First, some factors that are potentially associated with Co-MS were not collected due to the retrospective nature of this study. We selected the potentially associated factors based on the plausibility and availability (i.e., routinely collected information in clinical practice). Contrarily, numerous factors that were not collected in our study are reported as related factors with malnutrition and sarcopenia: history of hospitalisation, poor self-perceived health, loss of interest in life, polypharmacy, Parkinson’s disease, and constipation were associated with malnutrition [51,52]; nutrient intake, inactivity, disease, and iatrogenic factors potentially cause sarcopenia [53]. These factors can raise the risk for malnutrition and sarcopenia both before and after hospital admission and could be associated with Co-MS. Second, we could not conclude any causal relationship between Co-MS and potentially associated factors due to the cross-sectional design. Third, physical performance for diagnosing sarcopenia could not be performed because many study patients with cerebrovascular disease had poor physical performance or were unable to undergo testing due to disabilities such as hemiplegia. Appropriate methods for assessing physical performance in geriatric rehabilitation patients have not yet been established and require further investigation. Fourth, data on comorbidities relied on the diagnosis by medical doctors recorded on the electrical medical chart. Diseases that were not subject to treatment during rehabilitation (e.g., asymptomatic chronic heart failure), or were difficult to diagnose due to disabilities (e.g., cognitive impairment in the patients with aphasia) can be undiagnosed or misdiagnosed. Therefore, the data on CCI scores might be lowered.

## 5. Conclusions

The results of our cross-sectional study reveal that malnutrition and sarcopenia coexisted in 23.5% of the geriatric rehabilitation patients. Furthermore, a longer interval between disease onset and admission to rehabilitation wards, hospital-associated deconditioning, and poor swallowing function were potentially associated factors for Co-MS. Healthcare professionals should be aware of these factors in order to detect the geriatric rehabilitation patients who are at risk of both malnutrition and sarcopenia and to provide appropriate treatments. Further cohort studies will be needed to clarify the effects of the Co-MS on the functional outcomes for geriatric rehabilitation patients.

## Figures and Tables

**Table 1:** Characteristics of 601 patients admitted to convalescent rehabilitation wards.

	All (*n* = 601)	
Age, years	80	(72, 86)
Female sex, *n* (%)	355	(59.1)
Reason for admission, *n* (%)		
Cerebrovascular	425	(70.7)
Orthopaedics	164	(27.3)
Hospital-associated deconditioning	12	(2.0)
Onset–admission interval, days	21	(15, 28.5)
Charlson comorbidity index score ^a^	0	(0, 2)
Score ≥ 1, *n* (%)	274	(45.6)
Pre-morbid functional dependency ^b^, *n* (%)	155	(25.8)
Functional Independence Measure-admission ^c^		
Total	75	(47, 92)
Motor	50	(27, 64)
Cognitive	24	(16, 31)
Food Intake LEVEL Scale score ^d^	9	(8, 10)
Revised Oral Assessment Guide score ^e^	13	(11, 14)
Normal oral status, *n* (%)	18	(3.0)
Mild to moderate oral problems, *n* (%)	267	(44.4)
Severe oral problems, *n* (%)	316	(52.6)
Type of nutrition care, *n* (%)		
Oral intake	540	(89.9)
Oral intake + enteral nutrition	14	(2.3)
Enteral nutrition	47	(7.8)

Values are median (interquartile range), unless specified otherwise. ^a^ An indicator for the number of comorbidities which ranges from 0 to 24: a higher score indicates having more severe and/or more number of comorbidities. ^b^ *n* = 595 (six patients had missing values). Confirmed by the pre-morbid certification of public long-term care insurance. Patients who were certificated as “long-term care” (level 1 to 5) were identified as having pre-morbid functional dependency. ^c^ An indicator for activities of daily living, which ranges from 18 to 126 (motor score ranges from 13 to 91, cognitive score ranges from 5 to 35): a higher score indicates better ability to perform activities of daily living. ^d^ An indicator for swallowing dysfunction which ranges from 1 to 10: a higher score indicates better swallowing function. ^e^ An indicator for oral function/hygiene which ranges from 8 to 24: total scores of 8, 9 to 12 and 13 to 24 mean “normal oral status” (i.e., no oral problems), “mild to moderate oral problems”, and “severe oral problems”, respectively.

**Table 2:** Prevalence of malnutrition, sarcopenia, and coexistence of malnutrition and sarcopenia (Co-MS) in 601 patients admitted to the convalescent rehabilitation wards.

	All (*n* = 601)	
SMI, kg/m^2^, mean (SD)	5.6	(1.2)
Low SMI, *n* (%) ^a^	442	(73.5)
Maximum handgrip strength, kg	16.6	(9.9, 23.4)
Low hand grip strength, *n* (%) ^b^	443	(73.7)
Sarcopenia, *n* (%) ^c^	375	(62.4)
MUST score ^d^	0	(0, 1)
At risk of malnutrition, *n* (%)	263	(43.8)
BMI, kg/m^2^, mean (SD)	22.0	(3.5)
GLIM criteria-phenotype, *n* (%) ^e^		
Body weight loss	173	(28.8)
Low BMI ^f^	150	(25.5)
Low SMI ^a^	236	(39.3)
GLIM criteria -aetiology *n* (%) ^e^		
Reduced food intake/assimilation	167	(27.8)
Inflammation	35	(5.8)
Malnutrition, *n* (%) ^g^	174	(29.0)
Mild/moderate	60	(10.0)
Severe	114	(19.0)
Co-MS, *n* (%)	141	(23.5)

Values are median (interquartile range), unless specified otherwise. BMI, body mass index; Co-MS, coexistence of malnutrition and sarcopenia; GLIM, Global Leadership Initiative on Malnutrition; MUST, Malnutrition Universal Screening Tool; SD, standard deviation; SMI, skeletal muscle mass index. ^a^ Cut-off values: <7.0 kg/m^2^ for males and <5.7 kg/m^2^ for females [18]. ^b^ Cut-off values: <28 kg for males and <18 kg for females [18]. ^c^ Defined by fulfilling both low SMI and low handgrip strength based on the Asian Working Group for Sarcopenia criteria [18]. ^d^ Total score ranges from 0 to 6. A score of ≥1 was regarded as having a malnutrition risk. ^e^ Assessment was performed only for the patients with MUST scores of ≥1. ^f^ Asian-specific cut-off values: <18.5 kg/m^2^ for the patients aged <70 years, and <20.0 kg/m^2^ for patients aged ≥70 years [17]. ^g^ Defined by fulfilling ≥ 1 phenotypic criteria plus ≥ 1 aetiologic criteria of the GLIM criteria [17].

**Table 3:** Adjusted odds ratios for malnutrition, sarcopenia, and coexistence of malnutrition and sarcopenia (Co-MS) among 595 rehabilitation patients ^a^.

Variables	Adjusted Odds Ratio (95% Confidence Interval)		
	Malnutrition (*n* = 171, R^2^ = 0.18)	Sarcopenia (*n* = 372, R^2^ = 0.35)	Co-MS (*n* = 139, R^2^ = 0.23)
Age	1.02 (0.99, 1.05)	1.08 (1.05, 1.11) *	1.03 (1.00, 1.06)
Female sex	0.75 (0.49, 1.14)	1.18 (0.77, 1.81)	0.73 (0.46, 1.15)
Onset–admission interval	1.04 (1.02, 1.06) *	1.02 (1.00, 1.04)	1.04 (1.02, 1.06) *
Orthopaedics ^b^	0.94 (0.56, 1.58)	0.96 (0.59, 1.57)	1.35 (0.77, 2.35)
Hospital-associated deconditioning ^b^	3.63 (0.91, 14.4)	– ^c^	4.62 (1.13, 18.8) *
Pre-morbid functional dependency ^d^	1.23 (0.75, 1.99)	1.15 (0.67, 1.99)	1.32 (0.79, 2.20)
Charlson comorbidity index score	0.95 (0.79, 1.13)	1.08 (0.88, 1.32)	0.99 (0.82, 1.20)
Functional Independence Measure-motor	0.99 (0.98, 1.01)	0.98 (0.97, 1.00) *	0.99 (0.97, 1.00)
Functional Independence Measure-cognitive	1.01 (0.97, 1.04)	0.96 (0.93, 1.00) *	1.02 (0.98, 1.06)
Food Intake LEVEL Scale score	0.84 (0.75, 0.94) *	0.89 (0.76, 1.04)	0.83 (0.73, 0.93) *
Revised Oral Assessment Guide score	1.06 (0.97, 1.16)	1.01 (0.92, 1.11)	1.05 (0.95, 1.16)

* *p* < 0.05. ^a^ Adjusted for all potentially associated factors. Data on six patients were excluded because of missing values for pre-morbid functional independency. ^b^ Cerebrovascular disease as the reference. ^c^ Could not be calculated because all patients with hospital-associated deconditioning were sarcopenic. ^d^ Confirmed by the pre-morbid certification of public long-term care insurance.

**Table 4:** Sensitivity analysis of the adjusted odds ratios for malnutrition, sarcopenia, and coexistence of malnutrition and sarcopenia (Co-MS) among 531 rehabilitation patients who were able to measure handgrip strength ^a^.

Variables	Adjusted Odds Ratio (95% Confidence Interval)		
	Malnutrition (*n* = 139, R^2^ = 0.14)	Sarcopenia (*n* = 311, R^2^ = 0.33)	Co-MS (*n* = 109, R^2^ = 0.19)
Age	1.04 (0.99, 1.04)	1.09 (1.06, 1.12) *	1.03 (1.00, 1.06)
Female sex	0.93 (0.59, 1.47)	1.14 (0.74, 1.78)	0.87 (0.53, 1.45)
Onset–admission interval	1.04 (1.02, 1.06) *	1.02 (1.00, 1.04)	1.04 (1.02, 1.06) *
Orthopaedics ^b^	0.89 (0.52, 1.53)	0.99 (0.60, 1.63)	1.34 (0.75, 2.41)
Hospital-associated deconditioning ^b^	2.43 (0.55, 10.7)	– ^c^	2.98 (0.65, 13.6)
Pre-morbid functional dependency ^d^	1.25 (0.74, 2.10)	1.23 (0.70, 2.16)	1.32 (0.75, 2.31)
Charlson comorbidity index score	0.97 (0.80, 1.18)	1.08 (0.88, 1.33)	1.02 (0.83, 1.26)
Functional Independence Measure-motor	1.00 (0.98, 1.01)	0.99 (0.97, 1.00)	0.99 (0.97, 1.01)
Functional Independence Measure-cognitive	1.01 (0.97, 1.05)	0.97 (0.93, 1.00)	1.02 (0.98, 1.07)
Food Intake LEVEL Scale score	0.83 (0.73, 0.96) *	0.87 (0.73, 1.03)	0.80 (0.69, 0.92) *
Revised Oral Assessment Guide score	1.06 (0.96, 1.17)	1.01 (0.92, 1.12)	1.04 (0.94, 1.16)

* *p* < 0.05. ^a^ Adjusted for all potentially associated factors. Data on six patients were excluded because of missing value with pre-morbid functional independency. ^b^ Cerebrovascular disease as the reference. ^c^ Could not be calculated because all patients with hospital-associated deconditioning were sarcopenic. ^d^ confirmed by the pre-morbid certification of public long-term care insurance.

## Data Availability

The data presented in this study are available on request from the corresponding author when the ethics committee and the hospital where the study conducted permit.

## Supplementary Materials

The following are available online at https://www.mdpi.com/article/10.3390/nu13113745/s1, Table S1: Definition of malnutrition based on the Global Leadership Initiative on Malnutrition criteria considering the Asian-specific cut-off values for low BMI and low SMI, Table S2. Food Intake LEVEL Scale (FILS), Table S3: Revised Oral Assessment Guide (ROAG), Table S4: Prevalence of malnutrition, sarcopenia, and coexistence of malnutrition and sarcopenia (Co-MS) in 601 patients admitted to the convalescent rehabilitation wards by sex, Table S5: Crude odds ratios for malnutrition, sarcopenia, and coexistence of malnutrition and sarcopenia (Co-MS) among rehabilitation patients.

## References

[1] Cederholm T., Barazzoni R., Austin P., Ballmer P., Biolo G., Bischoff S.C., Compher C., Correia I., Higashiguchi T., Holst M. (2017). ESPEN guidelines on definitions and terminology of clinical nutrition. Clin. Nutr..

[2] Cruz-Jentoft A.J., Bahat G., Bauer J., Boirie Y., Bruyère O., Cederholm T., Cooper C., Landi F., Rolland Y., Sayer A.A. (2019). Sarcopenia: Revised European consensus on definition and diagnosis. Age Ageing.

[3] Nishioka S., Wakabayashi H., Momosaki R. (2018). Nutritional status changes and activities of daily living after hip fracture in convalescent rehabilitation units: A retrospective observational cohort study from the Japan Rehabilitation Nutrition Database. J. Acad. Nutr. Diet.

[4] Nishioka S., Wakabayashi H., Nishioka E., Yoshida T., Mori N., Watanabe R. (2016). Nutritional improvement correlates with recovery of activities of daily living among malnourished elderly stroke patients in the convalescent stage: A cross-sectional study. J. Acad. Nutr. Diet.

[5] Vandewoude M.F.J., Van Wijngaarden J.P., De Maesschalck L., Luiking Y.C., Van Gossum A. (2019). The prevalence and health burden of malnutrition in Belgian older people in the community or residing in nursing homes: Results of the NutriAction II study. Aging Clin. Exp. Res..

[6] Lelli D., Calle A., Pérez L.M., Onder G., Morandi A., Ortolani E., Colominas M., Pedone C., Inzitari M. (2019). Nutritional status and functional outcomes in older adults admitted to geriatric rehabilitations: The SAFARI Study. J. Am. Coll. Nutr..

[7] Wojzischke J., Van Wijngaarden J., Van Den Berg C., Cetinyurek-Yavuz A., Diekmann R., Luiking Y., Bauer J. (2020). Nutritional status and functionality in geriatric rehabilitation patients: A systematic review and meta-analysis. Eur. Geriatr. Med..

[8] Jang Y., Im S., Han Y., Koo H., Sohn D., Park G.Y. (2020). Can initial sarcopenia affect poststroke rehabilitation outcome?. J. Clin. Neurosci..

[9] Yoshimura Y., Wakabayashi H., Bise T., Tanoue M. (2018). Prevalence of sarcopenia and its association with activities of daily living and dysphagia in convalescent rehabilitation ward inpatients. Clin. Nutr..

[10] Matsushita T., Nishioka S., Taguchi S., Yamanouchi A. (2019). Sarcopenia as a predictor of activities of daily living capability in stroke patients undergoing rehabilitation. Geriatr. Gerontol. Int..

[11] Vandewoude M.F.J., Alish C.J., Sauer A.C., Hegazi R.A. (2012). Malnutrition-sarcopenia syndrome: Is this the future of nutrition screening and assessment for older adults?. J. Aging Res..

[12] Hu X., Zhang L., Wang H., Hao Q., Dong B., Yang M. (2017). Malnutrition-sarcopenia syndrome predicts mortality in hospitalized older patients. Sci. Rep..

[13] Sánchez-Rodríguez D., Marco E., Ronquillo-Moreno N., Miralles R., Vázquez-Ibar O., Escalada F., Muniesa J.M. (2017). Prevalence of malnutrition and sarcopenia in a post-acute care geriatric unit: Applying the new ESPEN definition and EWGSOP criteria. Clin. Nutr..

[14] Verstraeten L.M.G., Van Wijngaarden J.P., Pacifico J., Reijnierse E.M., Meskers C.G.M., Maier A.B. (2021). Association between malnutrition and stages of sarcopenia in geriatric rehabilitation inpatients: RESORT. Clin. Nutr..

[15] Sanchez-Rodriguez D., Marco E., Meza-Valderrama D., Dávalos-Yerovi V. (2020). Taking a step toward implementation of Global Leadership Initiative on Malnutrition (GLIM) criteria in geriatric rehabilitation. Eur. Geriatr. Med..

[16] Reijnierse E.M., De Van Der Schueren M.A.E., Trappenburg M.C., Doves M., Meskers C.G.M., Maier A.B. (2017). Lack of knowledge and availability of diagnostic equipment could hinder the diagnosis of sarcopenia and its management. PLoS ONE.

[17] Cederholm T., Jensen G.L., Correia M.T.I.D., Gonzalez M.C., Fukushima R., Higashiguchi T., Baptista G., Barazzoni R., Blaauw R., Coats A. (2019). The GLIM criteria for the diagnosis of malnutrition—A consensus report from the global clinical nutrition community. Clin. Nutr..

[18] Chen L.K., Woo J., Assantachai P., Auyeung T.W., Chou M.Y., Iijima K., Jang H.C., Kang L., Kim M., Kim S. (2020). Asian Working Group for Sarcopenia: 2019 consensus update on sarcopenia diagnosis and treatment. J. Am. Med. Dir. Assoc..

[19] Nishioka S., Wakabayashi H., Kayashita J., Taketani Y., Momosaki R. (2021). Predictive validity of the Mini Nutritional Assessment Short-Form for rehabilitation patients: A retrospective analysis of the Japan Rehabilitation Nutrition Database. J. Hum. Nutr. Diet.

[20] Nishioka S., Omagari K., Nishioka E., Mori N., Taketani Y., Kayashita J. (2020). Concurrent and predictive validity of the Mini Nutritional Assessment Short-Form and the Geriatric Nutritional Risk Index in older stroke rehabilitation patients. J. Hum. Nutr. Diet.

[21] Shiraishi A., Yoshimura Y., Wakabayashi H., Tsuji Y. (2018). Prevalence of stroke-related sarcopenia and its association with poor oral status in post-acute stroke patients: Implications for oral sarcopenia. Clin. Nutr..

[22] Miyai I., Sonoda S., Nagai S., Takayama Y., Inoue Y., Kakehi A., Kurihara M., Ishikawa M. (2011). Results of new policies for inpatient rehabilitation coverage in Japan. Neurorehabil. Neural Repair.

[23] Malnutrition Action Group (MAG) The “MUST” Explanatory Booklet. http://www.bapen.org.uk/pdfs/must/must_explan.pdf.

[24] Miyata S., Tanaka M., Ihaku D. (2013). The prognostic significance of nutritional status using malnutrition universal screening tool in patients with pulmonary tuberculosis. Nutr. J..

[25] Maeda K., Ishida Y., Nonogaki T., Mori N. (2020). Reference body mass index values and the prevalence of malnutrition according to the Global Leadership Initiative on Malnutrition criteria. Clin. Nutr..

[26] Nagano A., Maeda K., Shimizu A., Nagami S., Takigawa N., Ueshima J., Suenaga M. (2020). Association of sarcopenic dysphagia with underlying sarcopenia following hip fracture surgery in older women. Nutrients.

[27] Tanimoto Y., Watanabe M., Sun W., Hirota C., Sugiura Y., Kono R., Saito M., Kono K. (2012). Association between muscle mass and disability in performing instrumental activities of daily living (IADL) in community-dwelling elderly in Japan. Arch Gerontol. Geriatr..

[28] Nishioka S., Yamanouchi A., Matsushita T., Nishioka E., Mori N., Taguchi S. (2021). Validity of calf circumference for estimating skeletal muscle mass for Asian patients after stroke. Nutrition.

[29] Chumney D., Nollinger K., Shesko K., Skop K., Spencer M., Newton R.A. (2010). Ability of Functional Independence Measure to accurately predict functional outcome of stroke-specific population: Systematic review. J. Rehabil. Res. Dev..

[30] Kunieda K., Ohno T., Fujishima I., Hojo K., Morita T. (2013). Reliability and validity of a tool to measure the severity of dysphagia: The Food Intake LEVEL Scale. J. Pain Symptom Manag..

[31] Ribeiro M.T., Ferreira R.C., Vargas A.M., Ferreira e Ferreira E. (2014). Validity and reproducibility of the revised oral assessment guide applied by community health workers. Gerodontology.

[32] Tamiya N., Noguchi H., Nishi A., Reich M.R., Ikegami N., Hashimoto H., Shibuya K., Kawachi I., Campbell J.C. (2011). Population ageing and wellbeing: Lessons from Japan’s long-term care insurance policy. Lancet.

[33] Quan H., Li B., Couris C.M., Fushimi K., Graham P., Hider P., Januel J.M., Sundararajan V. (2011). Updating and validating the charlson comorbidity index and score for risk adjustment in hospital discharge abstracts using data from 6 countries. Am. J. Epidemiol..

[34] Gingrich A., Volkert D., Kiesswetter E., Thomanek M., Bach S., Sieber C.C., Zopf Y. (2019). Prevalence and overlap of sarcopenia, frailty, cachexia and malnutrition in older medical inpatients. BMC Geriatr..

[35] Cerri A.P., Bellelli G., Mazzone A., Pittella F., Landi F., Zambon A., Annoni G. (2014). Sarcopenia and malnutrition in acutely ill hospitalized elderly: Prevalence and outcomes. Clin. Nutr..

[36] Casaer M.P., Ziegler T.R. (2015). Nutritional support in critical illness and recovery. Lancet Diabetes Endocrinol..

[37] Kortebein P., Ferrando A., Lombeida J., Wolfe R., Evans W.J. (2007). Effect of 10 days of bed rest on skeletal muscle in healthy older adults. JAMA.

[38] McKendry J., Thomas A.C.Q., Phillips S.M. (2020). Muscle mass loss in the older critically ill population: Potential therapeutic strategies. Nutr. Clin. Pract..

[39] Sánchez-Rodríguez D., Marco E., Ronquillo-Moreno N., Maciel-Bravo L., Gonzales-Carhuancho A., Duran X., Guillén-Solà A., Vázquez-Ibar O., Escalada F., Muniesa J.M. (2019). ASPEN-AND-ESPEN: A postacute-care comparison of the basic definition of malnutrition from the American Society of Parenteral and Enteral Nutrition and Academy of Nutrition and Dietetics with the European Society for Clinical Nutrition and Metabolism definition. Clin. Nutr..

[40] Gariballa S., Alessa A. (2013). Sarcopenia: Prevalence and prognostic significance in hospitalized patients. Clin. Nutr..

[41] Foley N.C., Martin R.E., Salter K.L., Teasell R.W. (2009). A review of the relationship between dysphagia and malnutrition following stroke. J. Rehabil. Med..

[42] Fujishima I., Fujiu-Kurachi M., Arai H., Hyodo M., Kagaya H., Maeda K., Mori T., Nishioka S., Oshima F., Ogawa S. (2019). Sarcopenia and dysphagia: Position paper by four professional organizations. Geriatr. Gerontol. Int..

[43] Scherbakov N., Sandek A., Doehner W. (2015). Stroke-related sarcopenia: Specific characteristics. J. Am. Med. Dir. Assoc..

[44] Maeda K., Takaki M., Akagi J. (2017). Decreased skeletal muscle mass and risk factors of sarcopenic dysphagia: A prospective observational cohort study. J. Gerontol. Ser. A Biol. Sci. Med. Sci..

[45] Nishioka S., Yamasaki K., Ogawa K., Oishi K., Yano Y., Okazaki Y., Nakashima R., Kurihara M. (2020). Impact of nutritional status, muscle mass and oral status on recovery of full oral intake among stroke patients receiving enteral nutrition: A retrospective cohort study. Nutr. Diet.

[46] Wakabayashi H., Sashika H. (2014). Malnutrition is associated with poor rehabilitation outcome in elderly inpatients with hospital-associated deconditioning a prospective cohort study. J. Rehabil. Med..

[47] Zhao W.T., Yang M., Wu H.M., Yang L., Zhang X.-M., Huang Y. (2018). Systematic review and meta-analysis of the association between sarcopenia and dysphagia. J. Nutr. Health Aging.

[48] Sawa Y., Kayashita J., Nikawa H. (2020). Occlusal support is associated with nutritional improvement and recovery of physical function in patients recovering from hip fracture. Gerodontology.

[49] Nishioka S., Kokura Y., Okamoto T., Takayama M., Miyai I. (2021). Risk of weight loss in adult patients and the effect of staffing registered dietitians in Kaifukuki (convalescent) rehabilitation wards: A retrospective analysis of a nationwide survey. Healthcare.

[50] Maeda K., Shamoto H., Wakabayashi H., Akagi J. (2017). Sarcopenia is highly prevalent in older medical patients with mobility limitation: Comparisons according to ambulatory status. Nutr. Clin. Pract..

[51] O’Keeffe M., Kelly M., O’Herlihy E., O’Toole P.W., Kearney P.M., Timmons S., O’Shea E., Stanton C., Hickson M., Rolland Y. (2019). Potentially modifiable determinants of malnutrition in older adults: A systematic review. Clin. Nutr..

[52] Fávaro-Moreira N.C., Krausch-Hofmann S., Matthys C., Vereecken C., Vanhauwaert E., Declercq A., Bekkering G.E., Duyck J. (2016). Risk factors for malnutrition in older adults: A systematic review of the literature based on longitudinal data. Adv. Nutr..

[53] Cruz-Jentoft A.J., Sayer A.A. (2019). Sarcopenia. Lancet.

