# Supplementary material for: Prevalence and Associated Factors of Coexistence of Malnutrition and Sarcopenia in Geriatric Rehabilitation
Source: Nutrients. 2021 Oct 23;13(11):3745. doi: 10.3390/nu13113745 (PMC8620459; doi:10.3390/nu13113745)
Supplement: Supplementary file 1 [file nutrients-13-03745-s001.zip › nutrients-1360334-supplementary.pdf]

Table S1. Definition for malnutrition based on the Global Leadership Initiative on Malnutrition criteria in consideration with Asian-specific cut-off values for low BMI and low SMI [1,2–4] [a].

	Phenotypic Criteria			Etiologic Criteria	
	**Weight Loss**	**Low BMI (kg/m^2)**	**Low SMI (kg/m^2)**	**Reduced Food Intake or Assimilation**	**Inflammation**
Mild/ moderate malnutrition	5–10% within the past 6 months, or 10–20% beyond 6 months	17.0–<18.5 (<70 years) 17.8–<20 (≥70 years)	5.2–<7 for males 4.8–<5.7 for females	≤50% of ER > 1 week, or any reduction for >2 weeks, or any chronic gastrointestinal condition that can aggravate food assimilation or absorption	Suspected ongoing or history of inflammation by acute disease/injury or chronic disease
Severe malnutrition	>10% within the past 6 months, or >20% beyond 6 months	<17.0 (<70 years) [b] <17.8 (≥70 years) [b]	<5.2 for males [c] <4.8 for females [c]		

BMI, body mass index; SMI, skeletal muscle mass index; ER, energy requirement
a The patients fulfilled at least one phenotypic criterion and one etiologic criterion were diagnosed as malnutrition
b Ethnicity-specific cut-off values
c Minus 4 standard deviations from that of healthy young adults

Table S2. Food Intake LEVEL Scale (FILS) [5].

**No oral intake**	Definition
Level 1	No swallowing training is performed except for oral care.
Level 2	Swallowing training not using food is performed.
Level 3	Swallowing training using a small quantity of food is performed.
**Oral intake and alternative nutrition**	
Level 4	Easy-to-swallow food less than the quantity of a meal (enjoyment level) is ingested orally.
Level 5	Easy-to-swallow food is orally ingested in one to two meals, but alternative nutrition is also given.
Level 6	The patient is supported primarily by ingestion of easy-to-swallow food in three meals, but alternative nutrition is used as a complement.
**Oral intake alone**	
Level 7	Easy-to-swallow food is orally ingested in three meals. No alternative nutrition is given.
Level 8	The patient eats three meals by excluding food that is particularly difficult to swallow.
Level 9	There is no dietary restriction, and the patient ingests three meals orally, but medical considerations are given.
Level 10	There is no dietary restriction, and the patient ingests three meals orally (normal).

Swallowing training: Training conducted by an expert, well-instructed caregiver, or the patient himself/herself to improve the swallowing function.

Easy-to-swallow food: Food that is prepared so that it is easy to swallow even without mastication. For example, meat and vegetables are gelatinized or homogenized in a mixer.

Alternative nutrition: Non-oral nutrition such as tube feeding and drip infusion.

Food that is particularly difficult to eat: dry and brittle food, hard food, water, and so on.

Medical considerations: guidance, tests, examinations, and so on, for symptoms suggestive of swallowing disorders such as choking and the feeling of food remaining in the pharynx.

Table S3. Revised Oral Assessment Guide (ROAG) [6].

	Score		
	**1**	**2**	**3**
Voice	Normal	Deep or rasping	Difficulty talking or painful
Swallowing	Normal swallow	Some pain or difficulty on swallowing	Unable to swallow
Lips	Smooth and pink	Dry or cracked, and/or angular cheilitis	Ulcerated or bleeding
Teeth/dentures	Clean, no debris	Plaque or debris in local areas. Decayed teeth or damaged dentures	Plaque or debris generalised
Mucosa	Pink and moist	Dry and/or change in colour, red, blue-red of white	Very red, or thick, white coating. Blisters or ulceration with or without bleeding
Gingiva	Pink and firm	Oedematous and/or red	Bleeding easily under finger pressure
Tongue	Pink, moist and papillae present	Dry, no papillae present or change in colour (red or white)	Very thick white coating; blisters or ulceration
Saliva	No friction between the mouth mirror and mucosa	Slightly increased friction, no tendency for the mirror to adhere to the mucosa.	Significantly increased friction, the mirror adhering or tending to adhere to the mucosa

Table S4. Prevalence of malnutrition, sarcopenia, and coexistence of malnutrition and sarcopenia (Co-MS) in 601 patients admitted to the convalescent rehabilitation wards by sex.

	**Male (*n* = 246)**	**Female (*n* = 355)**
SMI, kg/m², mean (SD)	6.4 (0.9)	5.0 (0.9)
Low SMI, *n* (%) [a]	174 (70.7)	268 (75.5)
Maximum handgrip strength, kg	24.6 (17.2, 30.0)	13.7 (8.7, 17.9)
Low hand grip strength, *n* (%) [b]	174 (70.7)	269 (75.8)
Sarcopenia, *n* (%) [c]	143 (58.1)	232 (65.4)
MUST score [d]	0 (0, 1)	0 (0, 1)
At risk of malnutrition, *n* (%)	115 (46.7)	146 (41.1)
BMI, kg/m², mean (SD)	22.1 (3.5)	21.9 (3.6)
GLIM criteria-phenotype, *n* (%) [e]		
Body weight loss	90 (36.6)	83 (23.4)
Low BMI [f]	60 (24.4)	90 (25.4)
Low SMI [a]	100 (40.7)	136 (38.3)
GLIM criteria -aetiology *n* (%) [e]		
Reduced food intake/assimilation	77 (31.3)	90 (25.4)
Inflammation	19 (7.7)	16 (4.5)
Malnutrition, *n* (%) [g]	81 (32.9)	93 (26.2)
Mild/moderate	40 (16.3)	20 (5.6)
Severe	41 (16.7)	73 (20.6)
Co-MS, *n* (%)	63 (25.6)	78 (22.0)

Values are median (interquartile range), unless specified otherwise. BMI, body mass index; Co-MS, coexistence of malnutrition and sarcopenia; GLIM,

Global Leadership Initiative on Malnutrition; MUST, Malnutrition Universal Screening Tool; SD, standard deviation; SMI, skeletal muscle mass index.

[a] Cut-off values: <7.0 kg/m^2 for males and <5.7 kg/m^2 for females [18].

[b] Cut-off values: <28 kg for males and <18 kg for females [18].

[c] Defined by fulfilling both low SMI and low handgrip strength based on the Asian Working Group for Sarcopenia criteria [18].

[d] Total score ranges from 0 to 6. A score of ≥1 was regarded as having a malnutrition risk.

[e] Assessment was performed only for the patients with MUST scores of ≥1.

[f] Asian-specific cut-off values: <18.5 kg/m^2 for the patients aged <70 years, and <20.0 kg/m^2 for patients aged ≥70 years [17].

[g] Defined by fulfilling ≥1 phenotypic criteria plus ≥1 aetiologic criteria of the GLIM criteria [17].

Table S5. Crude odds ratios for malnutrition, sarcopenia, and coexistence of malnutrition-sarcopenia (Co-MS) among rehabilitation patients

Variables	Crude odds ratio (95% confidence interval)		
	Malnutrition (n = 174)	Sarcopenia (n = 375)	Co-MS (n = 141)
Age	1.02 (1.00, 1.05)*	1.11 (1.09, 1.14)*	1.04 (1.01, 1.06)*
Female sex	0.72 (0.51, 1.03)	1.36 (0.97, 1.90)	0.82 (0.56, 1.20)
Onset-admission duration	1.05 (1.03, 1.06)*	1.03 (1.01, 1.05)*	1.05 (1.03, 1.06)*
Orthopaedics [a]	0.50 (0.32, 0.78)*	0.65 (0.45, 0.94)*	0.66 (0.42, 1.05)
Hospital-associated deconditioning [a]	4.30 (1.27, 14.5)*	–[b]	6.17 (1.82, 20.9)*
Pre-morbid functional dependency [c]	1.66 (1.13, 2.46)*	3.43 (2.20, 5.35)*	2.14 (1.42, 3.21)*
CCI	1.13 (0.97, 1.32)	1.55 (1.30, 1.85)*	1.22 (1.04, 1.43)*
FIM-motor	0.97 (0.97, 0.98)*	0.96 (0.95, 0.96)*	0.97 (0.96, 0.98)*
FIM-cognitive	0.94 (0.92, 0.96)*	0.90 (0.88, 0.92)*	0.94 (0.91, 0.96)*
FILS	0.75 (0.69, 0.81)*	0.63 (0.55, 0.71)*	0.73 (0.67, 0.79)*
ROAG	1.19 (1.11, 1.28)*	1.26 (1.17, 1.36)*	1.21 (1.12, 1.30)*

CCI, Charlson comorbidity index; FIM, Functional Independence Measure; FILS, Food Intake LEVEL Scale; ROAG, Revised oral assessment guide.

*$P<0.05$

[a] Cerebrovascular disease as the reference

[b] Could not be calculated because all patients with hospital-associated deconditioning were sarcopenic

[c] Data on six patients were excluded because of missing values. Confirmed by the pre-morbid certification of public long-term care insurance.

References

1. Cederholm, T.; Jensen, G.L.; Correia, M.T.I.D.; Gonzalez, M.C.; Fukushima, R.; Higashiguchi, T.; Baptista, G.; Barazzoni, R.; Blaauw, R.; Coats, A.; et al. The GLIM criteria for the diagnosis of malnutrition—A consensus report from the global clinical nutrition community. *Clin. Nutr.* **2019**, *38*, 1–9, doi:10.1016/j.clnu.2018.08.002.
2. Miyata, S.; Tanaka, M.; Ihaku, D. The prognostic significance of nutritional status using malnutrition universal screening tool in patients with pulmonary tuberculosis. *Nutr. J.* **2013**, *12*, 1–5, doi:10.1186/1475-2891-12-42.
3. Maeda, K.; Ishida, Y.; Nonogaki, T.; Mori, N. Reference body mass index values and the prevalence of malnutrition according to the Global Leadership Initiative on Malnutrition criteria. *Clin. Nutr.* **2020**, *39*, 180–184, doi:10.1016/j.clnu.2019.01.011.
4. Nagano, A.; Maeda, K.; Shimizu, A.; Nagami, S.; Takigawa, N.; Ueshima, J.; Suenaga, M. Association of sarcopenic dysphagia with underlying sarcopenia following hip fracture surgery in older women. *Nutrients* **2020**, *12*, 1365, doi:10.3390/nu12051365.
5. Kunieda, K.; Ohno, T.; Fujishima, I.; Hojo, K.; Morita, T. Reliability and validity of a tool to measure the severity of dysphagia: The Food Intake LEVEL Scale. *J. Pain Symptom Manag.* **2013**, *46*, 201–206.
6. Ribeiro, M.T.; Ferreira, R.C.; Vargas, A.M.; Ferreira e Ferreira, E. Validity and reproducibility of the revised oral assessment guide applied by community health workers. *Gerodontology* **2014**, *31*, 101–110.
